# Comparative study on the effects of different feeding habits and diets on intestinal microbiota in *Acipenser baeri* Brandt and *Huso huso*

**DOI:** 10.1186/s12866-019-1673-6

**Published:** 2019-12-16

**Authors:** Guanling Xu, Wei Xing, Tieliang Li, Min Xue, Zhihong Ma, Na Jiang, Lin Luo

**Affiliations:** 1 Beijing Fisheries Research Institute, No. 18, Jiaomen Road, Fengtai district, Beijing, 100068 People’s Republic of China; 2 Feed Research Institute, Chinese Academy of Agricultural Sciences, No. 12, Zhongguancun south street, Haidian district, Beijing, 100081 People’s Republic of China

**Keywords:** Sturgeon, Intestinal microbiota, Different feeding habits, High-throughput sequencing

## Abstract

**Background:**

Siberian sturgeon (*Acipenser baeri* Brandt) and Beluga sturgeon (*Huso huso*) are two important commercial fish in China, and the feeding habits of them are very different. Diets and feeding habits are two significant factors to affect the gastrointestinal microbiota in fish. The intestinal microbiota has been reported to play a key role in nutrition and immunity. However, it is rarely reported about the relationship between the intestinal microbiota and feeding habits/diets on different Acipenseridae fish. This study is to comparative analysis of gut microbial community in Siberian sturgeon and Beluga sturgeon fed with the same diet/Beluga sturgeon fed with different diets in order to determine the effects of different feeding habits/diets on the fish intestinal microbiota.

**Results:**

According to the experimental objectives, BL and BH groups were Beluga sturgeon (*Huso huso*) fed with low fishmeal diet and high fishmeal diet, respectively. SH group represented Siberian sturgeon (*Acipenser baeri* Brandt) fed with the same diet as BH group. After 16 weeks feeding trial, the intestinal microbiota was examined by 16S rRNA high-throughput sequencing technology. On the phylum level, Proteobacteria and Bacteroidetes were significantly higher in BL group than BH group, and Cyanobacteria showed the opposite trend. Compared with BH group, Proteobacteria and Firmicutes were significantly increased in SH group, whereas Cyanobacteria were clearly decreased. At the genus level, *Pseudomonas* and *Citrobacter* in BL group were significantly higher comparing with BH group, while *Bacillus*, *Luteibacter*, *Staphylococcus* and *Oceanobacillus* was lower in BH group than SH group.

**Conclusions:**

Alpha and beta diversities indicated that the intestinal microflora were significant difference between Siberian sturgeon and Beluga sturgeon when they fed with the same diet. Meanwhile, Beluga sturgeon fed with low fishmeal diet can increase the species diversity of intestinal microbiota than it fed high fishmeal diet. Therefore, feeding habits clearly affected the gastrointestinal microbiota of sturgeons. Moreover, the impact of changes in food on the gut microbiota of sturgeons should be taken into consideration during the process of sturgeon aquaculture.

## Background

It is well known that the intestinal tract of vertebrates is a very complex and dynamic ecosystem, which is colonized by a large and diverse microbial community [1]. The intestinal microbiota of vertebrates has been reported to play a key role in nutrition and immunity, such as stimulating the growth and development of the intestinal epithelium, preventing it from pathogen invasion, contributing to the digestion of complex nutrients, and synthesizing beneficial secondary metabolites [2, 3]. As an aquatic vertebrate, the intestinal microbiota of fish has major influences on growth, health and development of fish [3, 4]. The stability of the intestinal microbiota is not only an extremely important factor in the inhibiting colonization of the intestine by pathogens, but also an essential factor for feed digestion [5, 6]. That is, investigation on the intestinal microbiota of fish may reveal intestinal development, homeostasis and the characterization of protection [2].

The composition of intestinal microbiota was impacted by many endogenous and exogenous factors, such as species, lifestyle, feeding habit, diet, nutritional status, living conditions, and so on [1, 3, 7]. Among environmental factors, dietary factors to a large extent affected the structure and abundance of intestinal bacterial community in fish [8]. Moreover, previous study indicated that the intestinal microbiota of omnivorous *Carassius cuvieri* showed the higher diversity than those of carnivorous individuals, which means that feeding habit was also the significant factor to affect intestinal microbiota composition [9]. Therefore, it is crucial to understand how such factors may influence the gut microbiota, with a view to regulate and control the bacterial community.

Sturgeon is the common name used for fish belonging to the Acipenseridae family that includes 27 species, such as *Acipenser*, *Huso*, *Scaphirhynchus* and *Pseudoscaphirhynchus* [10–12]. Siberian sturgeon (*A. baeri* Brandt) and Beluga sturgeon (*H. huso*) are two important species for aquatic farming in China not only for caviar, but also for meat [13, 14]. Siberian sturgeon belongs to the genus of *Acipenser*, and they have a wide range of food, which mainly feed on benthic animals, including chironomus larvae, mollusks, worms, crustaceans and small fish [15]. Besides, the previous study reported that Siberian sturgeon fed total plant-based diets with balance of essential amino acid (EAA) could maintain normal growth performance, which means that although Siberian sturgeon was the omnivorous fish, they also biased towards vegetarian diet [16]. Being carnivorous, Beluga sturgeon was come from the *Huso* genus and has the higher protein requirement to satisfy tissue growth and maintenance [17]. Meanwhile, there was evidence showed that replacing fishmeal with soybean meal without lactic acid significantly reduced growth performances of beluga, which further suggested that animal protein plays a key role in beluga diets due to their feeding habits [18]. The phylogenetic relationship between Siberian sturgeon and Beluga sturgeon is very close, because they belong with the same family. However, their diets are different, which may be due to their different gut microbiota. At present, the research is very limited about on the difference of intestinal microbiota between Siberian sturgeon and Beluga sturgeon. In order to promote the healthy cultivation of these two important aquaculture species, it is necessary to pay attention and study the intestinal bacterial communities and the factors affecting the composition and stability of the microbiota. Therefore, the present study was made to determine the intestinal bacterial communities of Beluga sturgeon fed two diets with different fishmeal levels, and to compare the intestinal microbiota of Siberian sturgeon and Beluga sturgeon fed with the same diet in the same environment, respectively.

## Results

### Analysis of 16S rRNA sequencing results

Six samples were taken from each group, but only 5, 5 and 3 samples from BL, BH and SH groups respectively accorded with the requirements of library construction, because the rest samples of PCR products were no purpose bands or low concentrations. The sequencing results were treated by a series of purification and filtration processes. Finally, a total of 246,700 sequences were obtained from the 13 samples, and the effective sequences of 13,740 to 28,143 were collected from each sample. The filtered sequencing data of each sample were counted in Additional file 1: Table S1. The proportion of effective tags among Raw_Tags was in the 60 to 80% range at each sample. After quality control filtration, the reads sequences within the corresponding length range of each sample were calculated. Distribution of the effective sequence length is shown in Additional file 1: Figure S1. 400 bp to 440 bp were the most effective sequence length distribution region.

**Fig. 1:**
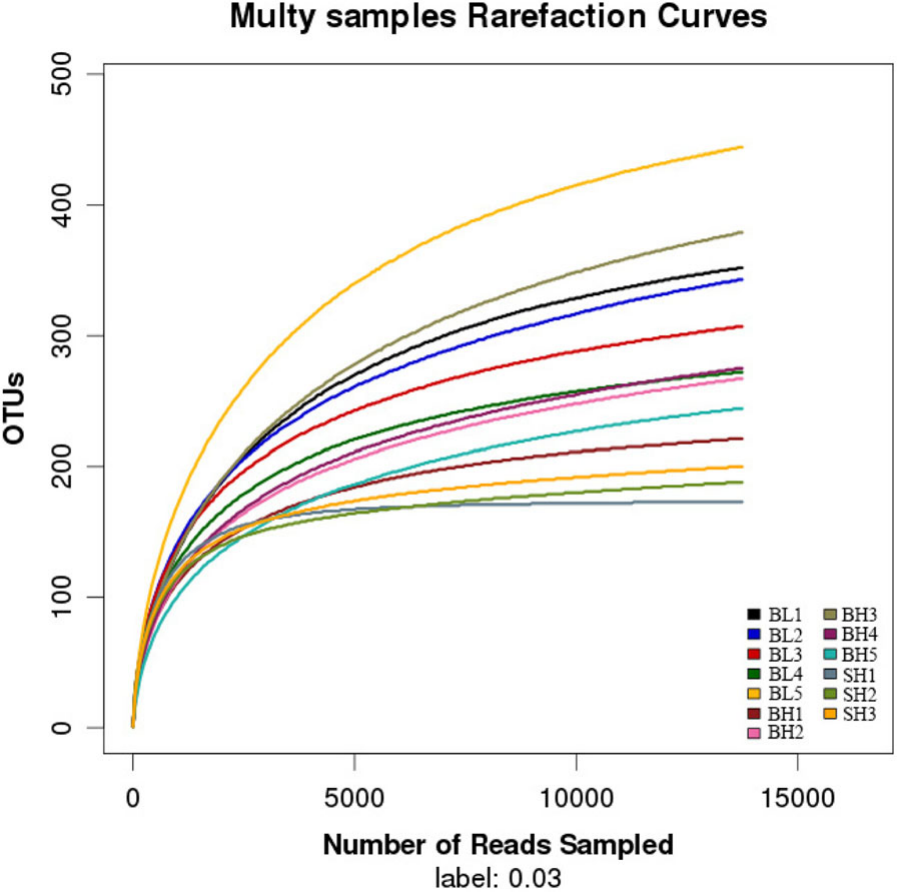
Rarefaction curve. The x-coordinate is the number of sequences sampled and the y-coordinate is the number of observed OTUs. Each curve in the graph represents a sample, which is labeled with a different color. The number of OTUs increases with the sequencing depth. When the curve becomes stable, the number of detected OTUs does not increase with the expansion of extracted data, indicating a time when the amount of sequencing data is reasonable

The sequences number of each sample OTU was distributed in the 97% sequence similarity threshold. Meanwhile, the classification information for each species corresponding to each OTU was also obtained by comparing the OTU representative sequences with a microbial reference database. The current results showed that the detected bacteria can be classified into 13 phyla, 21 classes, 41 orders, 77 families, and 120 genera. The composition of each sample community was calculated at phylum, class, order, family, genus and species levels, respectively. Additional file 1: Table S2 was showed the number of each species at different levels, and Additional file 1: Table S3 was recorded the total number of OTUs covered by each sample in their subordinate levels. The number of OTUs increased with the depth of sequencing, which was confirmed by the dilution curves of the OTUs measured in this study. The amount of sequencing data is reasonable because the final curve became stable (Fig. 1).

### Comparison of core intestinal microbiota in sturgeons from three experimental groups

**Fig. 2:**
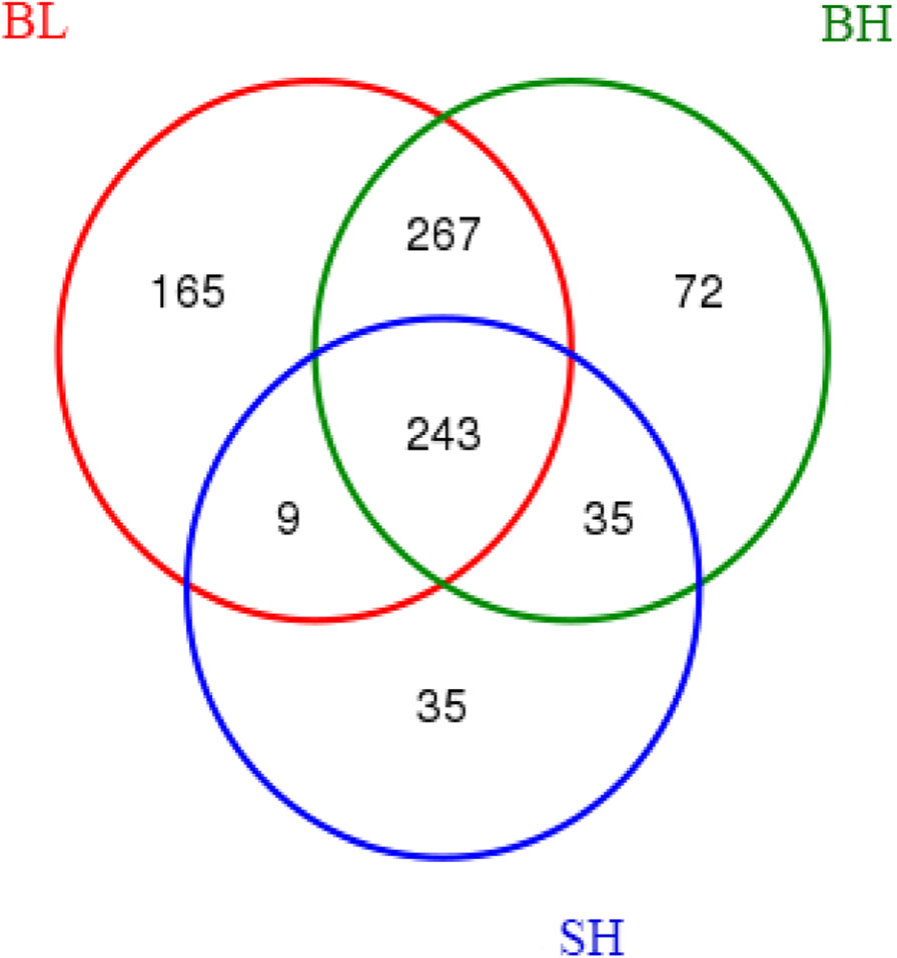
Venn diagram. The Venn diagrams show the numbers of OTUs (97% sequence identity) that were shared or not shared by all sturgeons in BL, BH and SH groups, respectively, depending of overlap

**Fig. 3:**
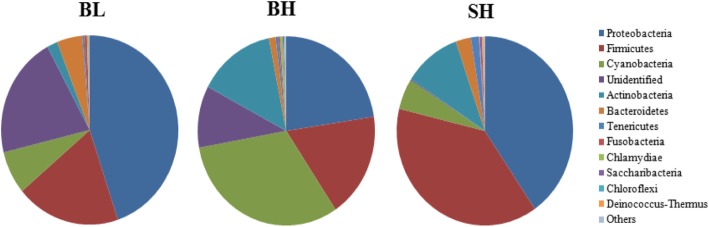
Pie charts. The pie diagram shows the 20 most abundant taxa (calculated over the combined dataset) in BL, BH and SH

The core intestinal microbiota of the sturgeons in three experimental groups was shown by Venn diagrams (Fig. 2). The core microflora of sturgeons meant the common bacterial populations in three experimental groups. All sturgeons in three experimental groups were shared 243 OTUs, while sturgeons in BL and BH groups were shared 510 OTUs, and 278 OTUs for sturgeon in BH and SH groups (Fig. 2). The main bacterial phyla in the intestines of three groups’ sturgeons are shown in Fig. 3. Proteobacteria, Firmicutes and Cyanobacteria were three dominant phyla in BL and BH groups, while SH group was Proteobacteria, Firmicutes and Actinobacteria. The core intestinal microflora in fish from three experimental groups were the same, but their relative abundances were different. The top five core intestinal microflora at phyla in BL group showed the order from high to low was Proteobacteria, Firmicutes, Cyanobacteria, Bacteroidetes and Actinobacteria (*P* < 0.05), whereas the core intestinal microflora in BH groups showed the difference with the order of Cyanobacteria>Proteobacteria>Firmicutes>Actinobacteria>Bacteroidetes. And the order from high to low of core intestinal microflora in SH group was Proteobacteria, Firmicutes, Actinobacteria, Cyanobacteria, and Bacteroidetes.

**Fig. 4:**
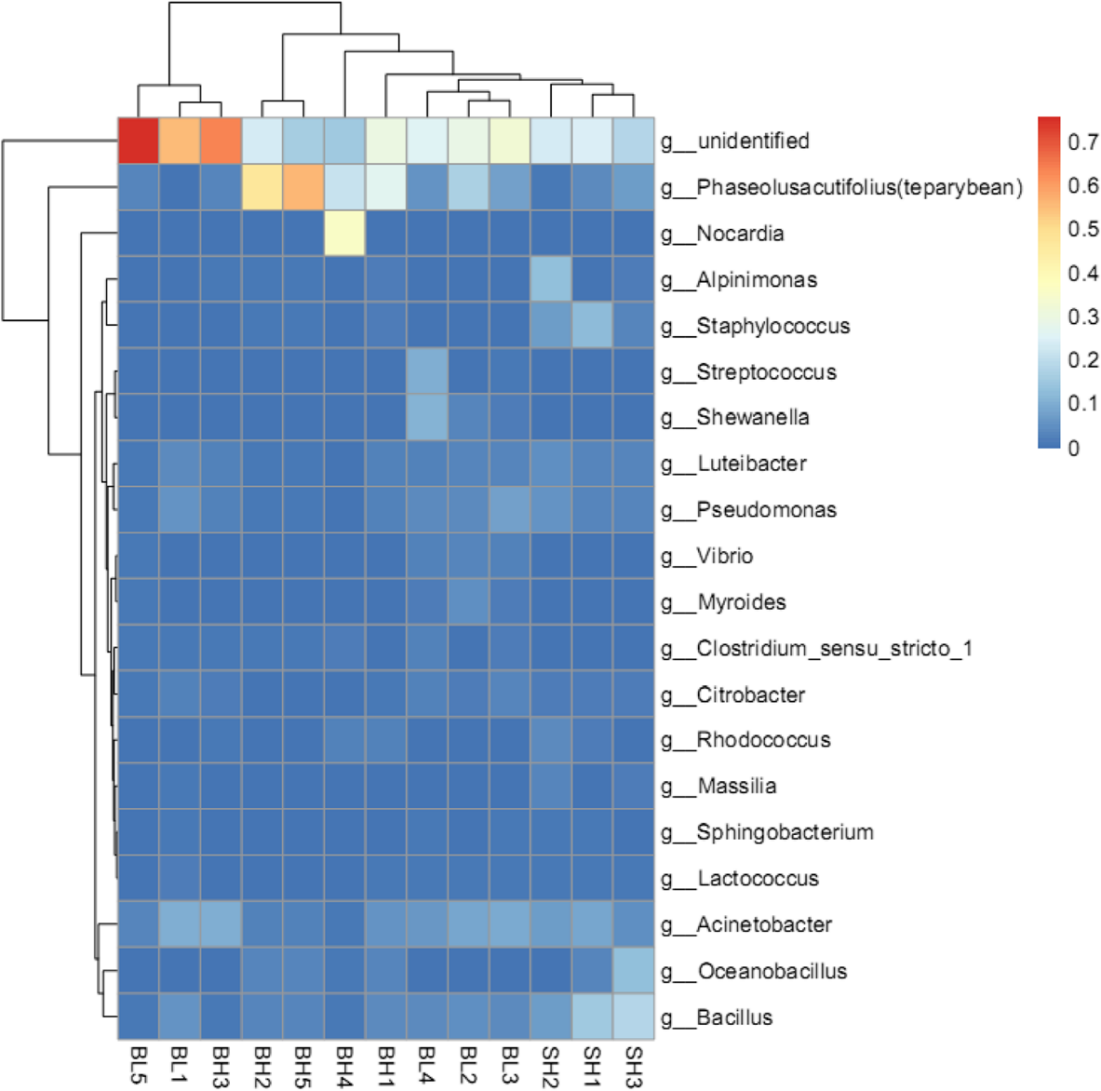
Heatmap showing richness of species at each level. The corresponding values of the heatmap are the Z values obtained by normalizing the relative abundance of species on each row. The color gradient from blue to red indicates a low to high relative abundance. The vertical clustering indicates the similarity in the richness of different species among different samples. The closer of distance between two species, the shorter of the branch length, indicated greater similarity in richness between the two species. Horizontal clustering indicates the similarity of species richness in different samples. Similarly, the closer the distance between two samples, the shorter the branch length, indicating greater similarity in richness of species between the two samples

A heatmap (Fig. 4) is a graphical representation that uses a system of colored gradients to represent the size of values in a data matrix, and the cluster data are also expressed in heatmap according to species or the abundance similarity of samples [19]. In order to reflect the similarities and differences between multiple sample communities, high-abundance and low-abundance species are clustered by color gradient and similarity [20]. Based on the species composition and relative abundance of each sample, a heatmap analysis was performed to extract the species at each taxonomic level [20]. Mapping was achieved using R language tools, and a heatmap cluster analysis was performed at the levels of the phylum, class, order, family, genus, and species, respectively [20]. The result was found in Fig. 4 that the vertical clustering between BH and SH groups showed the long branch length, which indicated the richness of intestinal microbiota between two groups was clearly different. Similarly, BL and BH groups have a certain degree of similarity in richness, because the short branch length was found in BL and BH groups at Fig. 4. In addition, the relative abundance of intestinal flora at genus level among BL, BH and SH groups were also showed in Fig. 4. The major intestinal microflora at genus level in BL group were *Acinetobacter*, *Pseudomonas*, *Bacillus*, and so on, as same as the BH group, while the SH group included *Bacillus*, *Staphylococcus* and *Acinetobacter* intestinal microflora at genus level.

### Alpha diversity analysis of microbial communities in sturgeons from three experimental groups

Alpha diversity indices mainly include Chao1, Observed_species, Shannon, PD_whole_tree, and Good’s coverage, which can response to the richness and diversity of a single sample species [20]. The Chao1 index measures the richness of species (i.e., the number of species), whereas the Shannon index measure the diversity of species [20]. Observed_species shows the number of OTU was observed with the increase of sequencing depth. PD_whole_tree refers to the number of species observed, reflecting the abundance of the colony. Good’s coverage reflects the completeness of the sequencing. In this study, 99% good’s coverage indicated that the most bacterial species present in the sample had been detected.

The alpha diversity results of the gut microbiota in sturgeons from three experimental groups were showed in Table 1. A total of 826 OTUs were obtained at the 97% similarity level. There was a significant difference in the OTUs, Chao1, Good’s coverage, Observed species and Shannon indices between the BH and SH groups (*P* < 0.05), but no significant difference was found in the PD whole tree of the two groups (*P* > 0.05). The result showed that the alpha diversity indices were considered to be significantly different between Siberian sturgeon and Beluga sturgeon when they fed with the same diet (*P* < 0.05).

**Table 1:** Number of reads, reads assigned to OTUs, good’s coverage and alpha diversity indices of intestinal microbiota composition in sturgeons from three experimental groups

	BL	BH	SH
OTUs	357.2 ± 32.27^b^	314.4 ± 30.14^b^	191.0 ± 9.64^a^
Chao1	390.05 ± 28.66^b^	328.61 ± 39.76^b^	214.21 ± 19.3^a^
Good’s coverage	0.994 ± 0.0005^a^	0.995 ± 0.0010^a^	0.998 ± 0.0007^b^
Observed species	335.32 ± 26.36^b^	271.92 ± 27.92^b^	186.47 ± 7.84^a^
PD whole tree	108.49 ± 18.45^b^	58.67 ± 23.33^ab^	18.67 ± 1.01^a^
Shannon	5.62 ± 0.08^b^	4.37 ± 0.27^a^	5.41 ± 0.02^b^

Note: means in the same row with different superscripts are significantly different (*P* < 0.05)

Meanwhile, although the most alpha diversity indice were no significant difference between BL and BH groups (*P* > 0.05), but the Shannon index was obviously higher in BL group than that in BH group (*P* < 0.05), which pointed that the species diversity of the intestinal microbiota was enhanced when Beluga sturgeon fed with the low fishmeal diet.

### Beta diversity analysis of microbial communities in sturgeons from three experimental groups

**Fig. 5:**
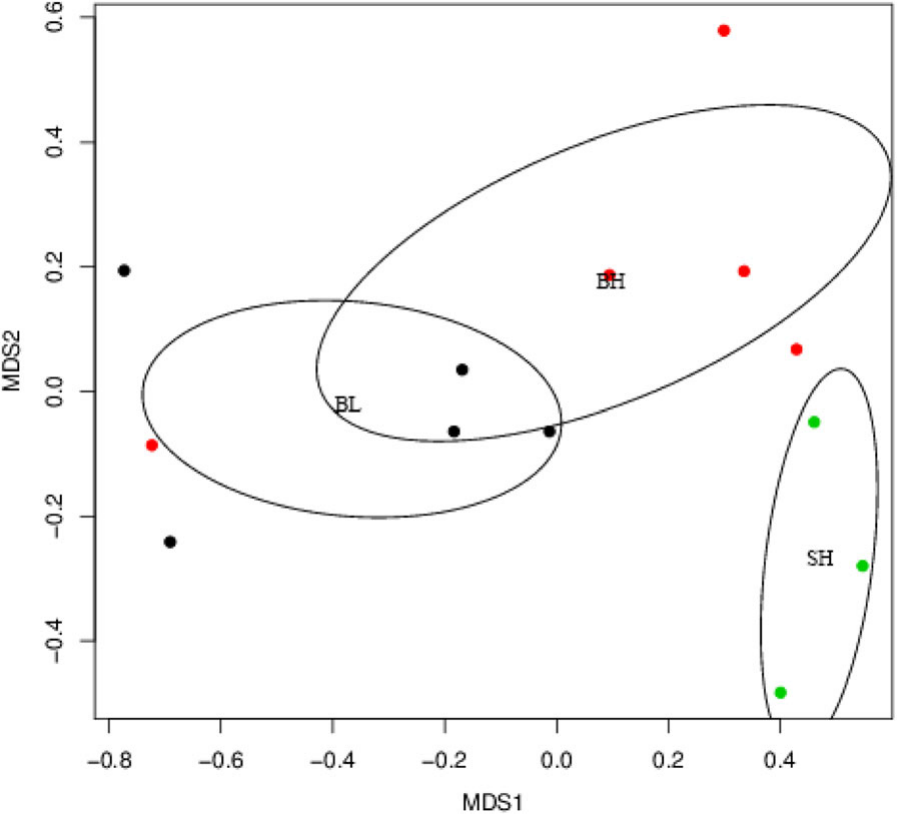
NMDS analysis. Different groups in the figure are represented by points in different colors (black: BL; red: BH; green: SH), and an eclips is made for the same group sample with biological repetition

Non-metric multidimensional scaling (NMDS) (Fig. 5) is a data analysis method that simplifies research objects (samples or variables) in multidimensional space to low-dimensional space for positioning, analysis and classification, while retaining the original relationship between objects. It is applicable to the case that the exact similarity or heterogeneity data between the research objects cannot be obtained, but only the hierarchical relationship data between them can be obtained. Its basic feature is to treat the data of similarity or dissimilarity between objects as a monotone function of the distance between points, and replace the original data with a new data column of the same order for the metric multidimensional scale analysis on the basis of maintaining the original data order relationship. In other words, when the data is not suitable for the direct multidimensional scaling analysis of variable type, the variable transformation is carried out and then the multidimensional scaling analysis of variable type is adopted. For the original data, it is called non-metric multidimensional scaling analysis. Its characteristics are reflected in multi-dimensional space in the form of points according to the species information contained in the sample, and the degree of difference between different samples is reflected by the distance between points, and finally the spatial locus map of the sample is obtained.

The closer the distance between sample points, the higher the similarity. Generally speaking, the samples within the same circle meant the difference between samples was not obvious, while the sample points within circles with no intersection indicated that there was significant difference between the samples. As shown in Fig. 5, the circles were intersect between BL and BH groups, but no intersection was found in BH and SH groups, which indicated the beta diversity between BH and SH groups was significant difference, whereas BH and BH groups was not.

### Analysis of the differences in intestinal microbiota among sturgeons in three experimental groups at the phylum and genus levels

**Fig. 6:**
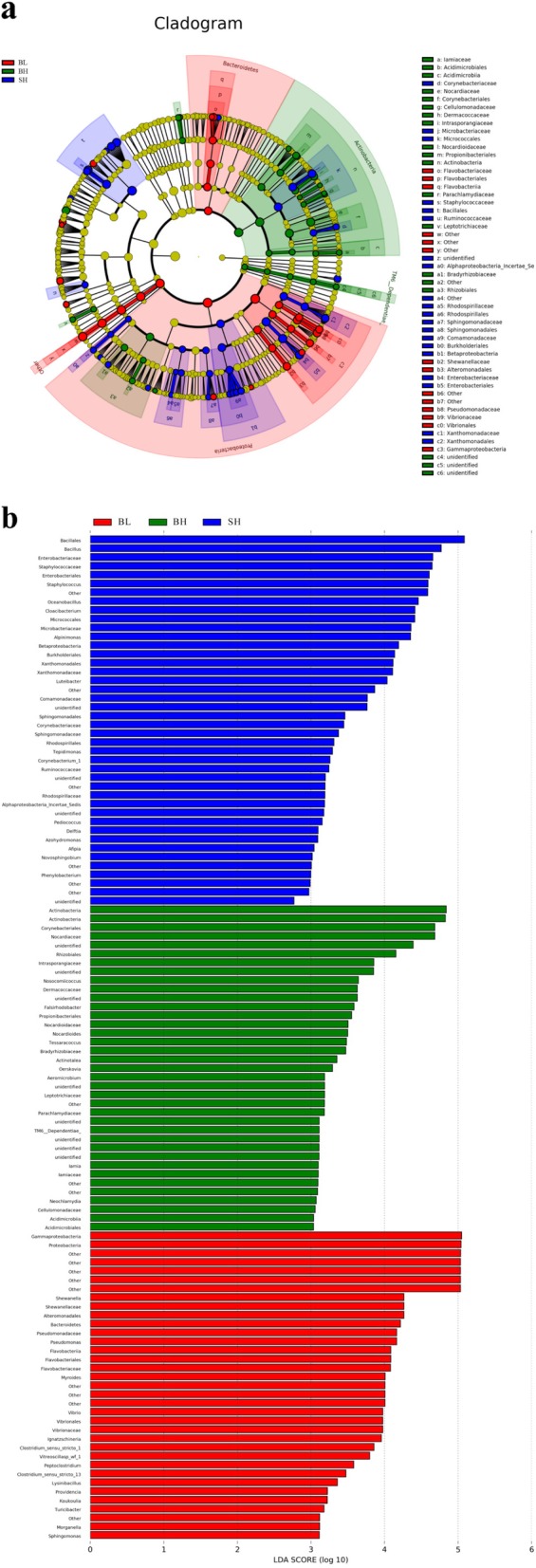
Taxonomic cladogram obtained from LEfSe analysis of 16S rRNA sequencing (**a**). Only taxa with LDA score > 2 are shown (**b**). **a** The circles from the inside to the outside of the evolutionary tree represent the classification from the phylum to the species level. Each small circle on a different classification level represents a classification below that level, and the diameter of the small circle is proportional to the relative abundance. Species with no significant differences are all represented by the color yellow, whereas the other significant different species are colored according to the group with the highest abundance to which the species belong. Different colors indicate different groups, and the nodes of different colors indicate the microorganisms that play an important role in the group represented by the color. **b** shows the species with significant differences of abundance in different groups, and the length of the histogram represents the influence size of the species with significant differences

LEfSe (Linear discriminant analysis Effect Size) (Fig. 6) is an algorithm for high-dimensional biomarker discovery and explanation that identifies bacteria of each level of phylum, class, order, family, or genus characterizing the differences among BH, BL and SH three groups (Fig. 6). The cladogram (Fig. 6a) showed differences in 102 taxa among fish in BL, BH and SH groups. BL fish (enrichment in the Gammaproteobacteria and Proteobacteria) have no similar to BH fish, with a lot of changes occurring in the BH group such as enrichment in Actinobacteria, Corynebacteria and Norynebacteria (Fig. 6b). And the SH group was mainly enriched in Bacillales and *Bacillus* genus, which was clearly difference with BH group (Fig. 6b).

**Fig. 7:**
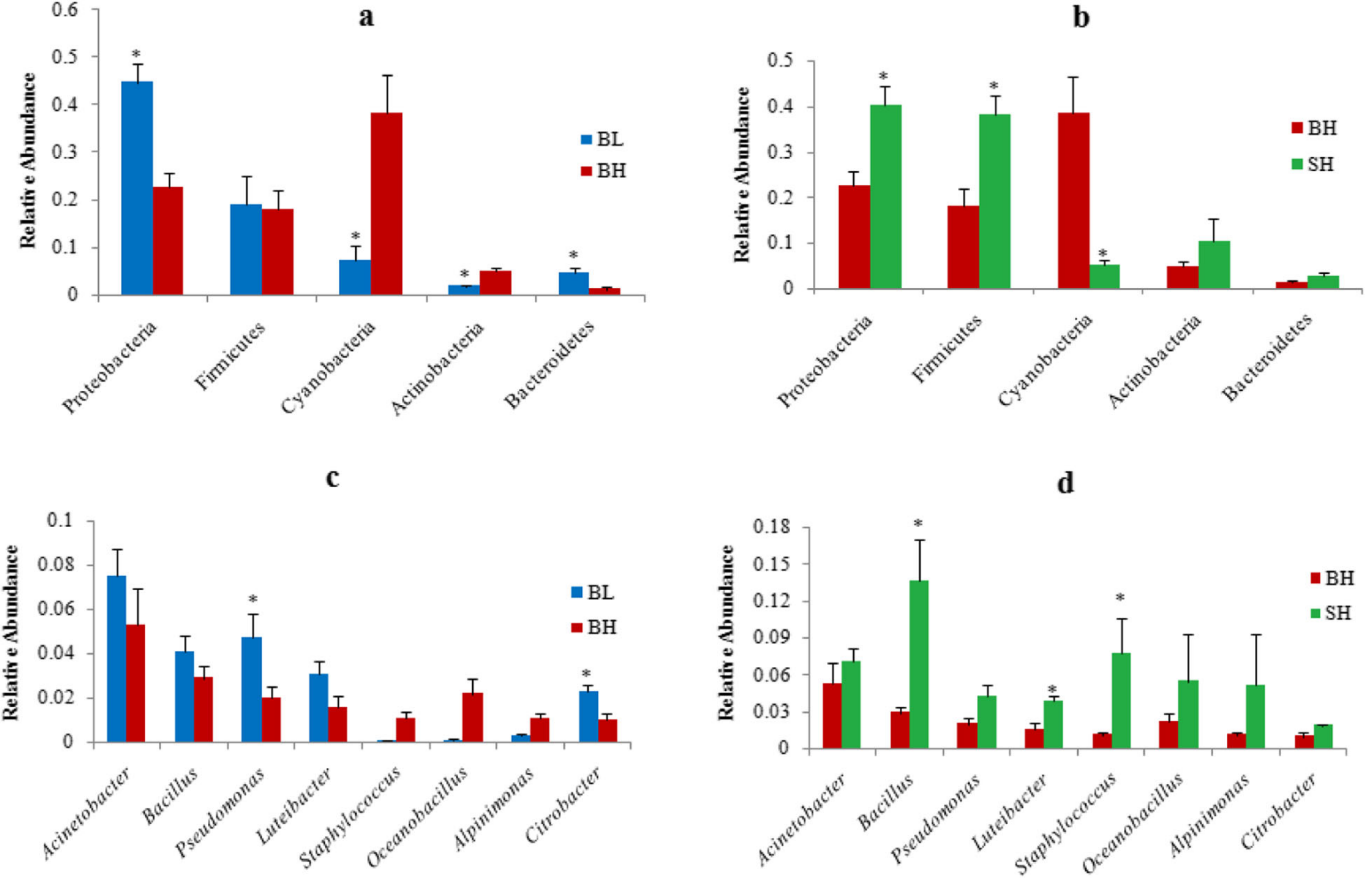
**a** and **c** Relative abundances (mean % SD) of five major bacterial phyla and eight major bacterial genera between BL and BH groups. **b** and **d** Relative abundance (mean % SD) of five major bacterial phyla and eight major bacterial genera between BH and SH groups. * means *P* < 0.05

In addition, Fig. 7 shows the relative abundance differences in phylum, family and genus levels among the three experimental groups. The relative abundance of Proteobacteria and Bacteroidetes in BL group sturgeons were significantly higher than that in BH group (*P* < 0.05), while the relative abundances of the Cyanobacteria phyla in BL group were significantly lower than in BH group (*P* < 0.05). The relative abundances of Firmicutes and Actinobacteria have no significant differences in both groups (*P* > 0.05) (Fig. 7a).

Compared with the sturgeons of BH group, the relative abundance of Proteobacteria and Firmicutes were significantly increased in sturgeons from SH group, whereas the relative abundance of Cyanobacteria were clearly decreased (*P* < 0.05). The Bacteroidetes and Actinobacteria relative abundance were no significant differences in sturgeons of BH and SH groups (*P* > 0.05) (Fig. 7b).

At the genus level, the relative abundances of *Pseudomonas* and *Citrobacter* in BL group were significantly higher compared with BH group (*P* < 0.05) (Fig. 7c), while the relative abundance of *Bacillus*, *Luteibacter*, *Staphylococcus*, and *Oceanobacillus* was lower in BH group than in SH group (*P* < 0.05) (Fig. 7d). Three groups did not show any significant differences in the relative abundances of *Alpinimonas* and *Acinetobacter* (*P* > 0.05).

## Discussion

With the in-depth study of intestinal microbiota in aquatic animals and the wide application of high-throughput sequencing technology, the progress was visible about the study on the relationship between gastrointestinal microecology and fish health [21]. The research of fish intestinal microbiota can help for understanding how gut microbial communities are assembled and how they impact host fitness. In the same environment, the present study was the first one to compare gut bacterial communities of Beluga sturgeon and Siberian sturgeon fed with the same diet and of Beluga sturgeon fed with two different diets by high-throughput sequencing methodology. Based on the high-throughput sequencing technology, thirteen samples from the intestine contents and mucosa in three groups analyzed the V3/V4 regions of the 16S rRNA gene.

The analysis results showed that 400 bp to 440 bp was a the most effective sequence length distribution region, and the amount of sequencing data in this study was reasonable because the number of OTUs increased and eventually leveled off with the sequencing depth increased (Figs. 1 and Additional file 1: Figure S1). As seen in Figs. 2 and 3, Proteobacteria, Firmicutes, Cyanobacteria, and Actinobacteria were the main intestinal microbiota composition of BH and SH groups when they fed with the same diet, which was accorded with the majority of studies on the sturgeon intestinal microbiota [22–24]. Noteworthily, the relative abundances of intestinal microbiota were completely different in the BH and SH groups, which showed that the intestinal microbiota was difference when the different Acipenserdae fish (Siberian sturgeon and Beluga sturgeon) fed with the same diets. In other words, this result further indicated that feeding habit is an important factor that affects gastrointestinal microbiota. Many previous studies reported that the intestinal microbiota was influenced by the host genotype [25, 26]. Similar results were discovered by Li et al. [27], who point out different species of Carp fed with a commercial fish food in the same earth pond have the difference bacterial communities in the intestines. Meanwhile, three eel species for investigating the autochthonous microbiome using 16S rDNA sequencing indicated that the composition of intestinal microbiome of eel was affected by the characteristics of different eel species [28]. In addition, the alpha and beta diversity indices of the intestinal microbiota between BH and SH groups were also significantly different, which also confirmed the above point of view that the richness and diversity of the intestinal microorganisms are indeed dissimilar in the different species of sturgeons although they fed with the same diet. This result was also consistent with Li et al. [29], who reported that feeding habits and genotype clearly affected the gastrointestinal microbiota of fish.

On the other hand, although the dominant phyla in BL and BH group were Proteobacteria, Firmicutes and Cyanobacteria, while their relative abundance of the dominant phyla was significant difference, which indicated that the same fish (Beluga sturgeon) fed different diets were able to affect the intestinal microbiota. Lv et al. [23] found that the relative abundances of intestinal microbiota were difference between the wild Kaluga (*Huso dauricus*) and cultured Kaluga sturgeon, and their predominant bacteria are Proteobacteria and Fusobacteria, respectively. Besides, there were reported that the microbiota composition of Atlantic Salmon (*Salmo salar*) was influenced by experimental diets when they fed with fishmeal, soybean meal or fermented soybean meal diets [30].

In addition, BL and BH groups had no obviously differences in the alpha and beta diversity indices of the intestinal microbiota except for Shannon index. The result hinted that the different diets had a little effect on the richness of intestinal flora in the same fish (Beluga sturgeon) within a certain period of time. Studies have shown that the normal dominant bacteria in the intestinal tract of aquatic animals are anaerobic bacteria, accounting for more than 99%, and aerobic bacteria and facultative anaerobic bacteria account for about 1% [31]. Due to the symbiosis between anaerobic bacteria and intestinal wall, the content of anaerobic bacteria was stable, while the aerobic bacteria can be free in the middle of the intestinal cavity, so the content of aerobic bacteria was a random fluctuation in the intestinal tract [32]. As a result, the total number of anaerobic bacteria in the intestinal tract of different fishes has little difference, while the total number of aerobic bacteria has great difference [33]. This may explain why the richness of intestinal flora in the same fish with different diets is the same. Interestingly, the Shannon index was significantly higher in BL group compared with BH group, which indicated that Beluga sturgeon fed with low fishmeal diets (fishmeal partially replaced by cottonseed protein) can enhance the diversity of intestinal microbiota. This result was similar with the earlier study on the northern snakehead (*Channa argus* Cantor, 1842), which showed that different dietary soybean meal substitutions significantly affected the intestinal microbiota composition [34]. Similar results were also found in other fish species, such as rainbow trout (*Oncorhynchus mykiss* Walbaum) [35], European sea bass (*Dicentrarchus labrax*) [36] and Atlantic Salmon (*S. salar*) [30].

At the phylum level, the richness of Proteobacteria and Bacteroidetes in the intestines of BL group was clearly higher than that of BH group, whereas for Cyanobacteria was exactly the opposite (Fig. 7a). Proteobacteria, as the most abundant in the fish ponds, participated in various biogeochemical processes (for example, carbon, nitrogen, and sulfur cycling) in aquatic ecosystems [37, 38]. Previous studies have indicated that Proteobacteria were one of the most abundant phyla in the intestinal samples of fish as well as in most mammals gut samples [39]. Some researchers have already verified that some bacteria of Proteobacteria in the gut of healthy fish may significantly contribute to the digestive function [3]. Bacteroidetes is responsible for the metabolism of steroids, polysaccharides, and bile acids, helping the host in the absorption of polysaccharides and the synthesis of protein [40, 41]. Moreover, Bacteroidetes was also typically enriched in other herbivores [42–44]. In other words, herbivores were more likely to accumulate more Bacteroidetes compared with carnivores. Hence, we may infer that the intestines of the BL group have the higher amount of Proteobacteria and Bacteroidetes might be because the fishmeal was partially replaced by cottonseed meal in the diet of the BL group.

The ratio of Firmicutes to Bacteroidetes ratio (the F/B ratio) is significantly correlated with obesity [45–48]. There were also reported that the F/B ratio of the gastrointestinal microbiota of carnivorous fishes were obviously higher than other fishes [29]. Coincidently, the result of this study showed that the F/B ratio of SH group was 13.8, while the F/B ratio in BH group was 14.6 (Fig. 7b), which indicated that Beluga sturgeon is actually bias carnivorous than the Siberian sturgeon. This result accorded with previous study, which reported that Siberian sturgeon was the omnivorous fish, they also biased towards vegetarian diet when they compared with beluga [16].

At the family and genus levels, the relative abundance of *Pseudomonas* and *Citrobacter* in BL group was significantly higher than those in BH group (*P* < 0.05) (Figs. 4, 6 and 7c). Previous studies have reported that *Citrobacter* was one of the important cellulose-degrading bacteria [49–51]. This point of view can explain our results that the abundance of *Citrobacter* was increased when Beluga sturgeon fed with low fishmeal in order to improve the digestion of cellulose. Similar results were reported by Liu et al. [52], who found that *Citrobacter* was one of the most abundant bacteria in the herbivorous fish. Wu et al. [53] also discovered that *Citrobacter* was one of the most abundant in grass carp samples. In addition, the previous work reported that the *Pseudomonas* was more frequently found in grass carp (*C. idella*) fed a soybean meal diet compared to a casein meal diet. The current study also showed that the abundance of *Pseudomonas* was enhanced in Beluga sturgeon when they fed with high vegetable protein diets. Therefore, the increase of these pathogenic bacteria may indicate that Beluga sturgeon might not be adapted to the low fishmeal diet very well, increasing the risk of intestinal infections.

Another result (Figs. 4, 6 and 7d) indicated that the relative abundance of *Bacillus*, *Luteibacter*, and *Staphylococcus* of the sturgeons in SH group was significantly higher than those in BH group on the family and genus levels (*P* < 0.05). The previous studies reported that the genus of *Bacillus*, as a typical probiotic, was used for enhancing host immunity and extracting nutrients consumed in fish diets [54, 55]. The function of *Bacillus* was able to improve the activity of digestive enzymes, enhance the immune system response, improve survival rate and confers disease resistance against pathogenic *Vibrio* species [56]. Moreover, *Bacillus* was also one of the important cellulose-degrading bacteria [49–51]. Hence, there were two points to contribute to the present result. On the one hand, Siberian sturgeons had the better colonization of *Bacillus* than Beluga sturgeon when both of them fed with the same diet. On the other hand, the increasing of *Bacillus* can improve the cellulose digestion in the Siberian sturgeon, which means that Siberian sturgeon had higher adaptability for vegetarian diet when they compared with Beluga sturgeon.

## Conclusions

In conclusion, Siberian sturgeon and Beluga sturgeon, as the two different species from the same family, were significantly different in feeding habits. When they fed with the same diets in the same environment at the present study did not result in similar intestinal bacteria, suggesting that the specific endogenous factors outweighed by far the environmental factors to mould the composition of microbiota [27]. Meanwhile, our results showed that Beluga sturgeon fed with the low fishmeal diets can increase the species diversity of intestinal microbiota compared with it fed with high fishmeal diets. Therefore, the impact of changes in food on the gut microbiota of the sturgeons should be taken into consideration during the process of sturgeon aquaculture.

## Methods

### Experimental design and diet formulation

Two isonitrogenous (410 g/kg crude protein) and isocaloric (19 MJ/kg) diets were formulated to contain two different fishmeal levels [high fishmeal: 250 g fishmeal/kg diet, FM250] and [low fishmeal: 100 g fishmeal/kg diet, FM100] [13]. The FM100 diet was supplemented with threonine, methionine, lysine and fish oil to keep the same EAA and highly unsaturated fatty acid (HUFA) composition with FM250 diet. The diets were made into sinking extruded pellets by an extruder (MY56X2A, MUYANG Group, Jiangsu province, China) in different pellet diameters (2.0 mm and 3.0 mm) according to fish size. All experimental diets were air-dried and stored at − 20 °C until use. Diets formulation and proximate compositions are shown in Table 2.

**Table 2:** Ingredient composition and proximate composition of experimental diets ^d^

Ingredient (g/kg diet)	FM 100	FM 250
Fish meal^a^	100	250
Soybean meal	230	250
Wheat flour	163.4	221
Krill meal	60	60
Wheat gluten	50	50
Brewer’s yeast	50	50
Soybean lecithin	20	20
Fish oil^a^	36.3	25
Soybean oil	17	24
Premix^b^	20	20
Cottonseed protein	190	0
65% Lysine	9	0
Methionine	1.5	0
Threonine	1.8	0
Ca(H_2_PO_4_)_2_	51	30
Proximate composition (g/kg)		
Crude protein	410	407
Crude fat	91	92
Moisture	80	94
Ash	95	79
Gross energy (MJ/kg)	19.1	19.1
Total phosphorus	16.1	16.3
Total calciums	14.7	15.3
Threonine	15.3	14.8
Methionine	7.7	7.4
Lysine	23.3	22.6
EPA^c^	5.5	5.3
DHA^c^	5.1	5.2

^a^ Fish meal and fish oil were produced in Peru and supplied by the International Fish Meal and Fish Oil Organization (IFFO, Hertfordshire, UK); Soybean meal, soybean oil and lecithin were supplied by YiHai Kerry Investment Company Limited, Shandong, China; Wheat flour were supplied by Guchuan Group, Beijing, China

^b^ Including vitamin premix (mg/kg diet): vitamin A 20; vitamin B_1_ 12; vitamin B_2_ 10; vitamin B_6_ 15; vitamin B_12_ 8; niacinaminde 100; ascorbic acid 1000; calcium pantothenate 40; biotin 5; folic acid 10; vitamin E 400; vitamin K_3_ 20; vitamin D_3_ 10; inositol 200; corn protein powder 150. Mineral premix (mg/kg diet): CuSO_4_·5H_2_O 10; FeSO_4_·H_2_O 300; ZnSO_4_·H_2_O 200; MnSO_4_·H_2_O 100; KIO_3_ (10%) 80; Na_2_SeO_3_ (10% Se) 67; CoCl_2_·6H_2_O (10% Co) 5; NaCl 100; Zeolite 16,138

^c^ *EPA* Eicosapentaenoic Acid, *DHA* Docosahexaenoic Acid

^d^ All diets were produced at National aquafeed safety evaluation station, Beijing, China, as extruded pellets

Three experimental groups were designed for this experiment including BL, BH and SH groups. BL and BH groups were Beluga sturgeon fed with low fishmeal diet (FM100) and high fishmeal diet (FM250), respectively. SH represented that Siberian sturgeon fed with high fishmeal diet (FM250) as same as BH group. The purpose of the design was to compare the difference of intestinal microbiota in Beluga sturgeon fed with two difference diets, and to analyze the composition of the intestinal microbial communities between Beluga sturgeon and Siberian sturgeon when they fed with the same diet.

### Rearing conditions and sample collection

Siberian sturgeon and Beluga sturgeon were purchased from Zhongketianli Aquatic science and technology Co. Ltd. (Beijing, China), where the feeding trial was also performed in a flow-through fish rearing system using recirculation water and under natural photoperiod conditions. Siberian sturgeon and Beluga sturgeon were acclimated to the flow-through fish rearing system and fed with a commercial fish food for 2 weeks before the trials. The specific parameters of system are as follows: temperature 18.0 ± 1.0 °C, dissolved oxygen > 7.5 mg/L, pH 8.1 ± 0.2 and nitrite < 0.1 mg/L, which ensured nearly constant and optimal water quality to fishes. Then, healthy sturgeon (37.5 ± 0.0 g) were randomly distributed into 9 net cages (capacity: 900 L) at the same cement pool. Triplicate replicate tanks were randomly assigned to three experimental groups (BL, BH and SH), and 60 fish were batch weighed and stocked in each tank. During the 16-week feeding period, fish were fed with the experimental diets to apparent satiation three times daily at 8:00, 13:00 and 18:00 respectively.

At the end of the feeding trial, all fish were fasted for 24 h before sampling. Two fish in each tank were randomly and quickly captured and anaesthetized with an overdose of 2-phenoxy ethanol solution. In order to reduce contamination, the surface of fish is rinsed with sterile distilled water firstly and wiped with 70% ethanol [27]. Then used the sterile scissors dissected the fish [27]. The whole intestinal tract with faecal was removed from abdominal cavity stored in − 80 °C immediately for the following intestinal microbiology analysis. All sampling operations were conducted on the ice [53]. At the end of the experiment, all remaining fish will continue to be farmed in Zhongketianli Aquatic science and technology Co. Ltd. until the fish grow up and the company has the ownership and right to use the fish.

### DNA extraction, purification and PCR amplification

Samples (intestinal content and water sediment) were thawed on ice, and then total bacterial DNA was extracted with Power Fecal DNA extraction kit (Mo Bio, USA) according to the manufacturer’s instructions [27]. Using the 0.8% agarose gels checked the purity and quality of the genomic DNA. And the DNA concentration was measured by using a fluorescence spectrophotometer (ES-2, Malcom, Japan). In order to avoid bias, all samples were extracted in duplicates, and the same sample extracts were mixed together [53, 57]. The extracted DNA was stored at − 80 °C until it was used for high-throughput sequencing [58].

In a word, the primer, 336F-806R (F: GTACTCCTACGGGAGGCAGCA; R: GTGGACTACHVGGGTWTCTAAT), was used to conduct PCR amplification of the 16S rRNA V3-V4 region. For each sample, 10-digit barcode sequence was added to the 5′ end of the forward and reverse primers (provided by Allwegene Company, Beijing). The PCR was carried out on an APPlied Biosystems GeneAmp PCR system 9700 using 50 μl reaction volumes, containing 5 μl 10 × Ex Taq Buffer (Mg^2+^ plus), 4 μl 12.5 mM dNTP Mix (each), 1.25 U Ex Taq DNA polymerase, 2 μl template DNA, 200 nM bar coded primers 336F and 806R each, and 36.75 μl ddH_2_O. Thermal cycling parameters were 94 °C for 2 min, followed by 30 cycles of 94 °C for 30 s, 57 °C for 30 s and 72 °C for 30 s with a final extension at 72 °C for 10 min. In order to mitigate reaction-level PCR biases, three PCR products per sample were mixed. The PCR products were purified using a QIAquick Gel Extraction Kit (QIAGEN, Germany), quantified using Real Time PCR, and sequenced at Allwegene Company, Beijing.

### High throughput sequencing analysis

At last, deep sequencing was performed on Miseq platform at Allwegene Company (Beijing). After the run, image analysis, base calling and error estimation were performed using Illumina Analysis Pipeline Version 2.6. The raw data were first screened and sequences were removed from consideration if they were shorter than 200 bp, had a low quality score (≤20), contained ambiguous bases or did not exactly match to primer sequences and barcode tags. Qualified reads were separated using the sample-specific barcode sequences and trimmed with Illumina Analysis Pipeline Version 2.6. And then the dataset were analyzed using QIIME software package (Quantitative Insights Into Microbial Ecology) [59]. The sequences were clustered into operational taxonomic units (OTUs) at a similarity level of 97%, to generate rarefaction curves and to calculate the richness and diversity indices [60, 61]. The Ribosomal Database Project (RDP) Classifier tool was used to classify all sequences into different taxonomic groups [62, 63]. Mothur software [64] was used to calculate the alpha diversity indexes of the purified samples to obtain the richness and diversity indices of the bacterial community (i.e., ACE, Chao1, Shannon, and Simpson). To examine the similarity between different samples, heatmap figures, venn diagrams and non-metric multidimensional scaling (NMDS) were used based on the OTU information from each sample using R, while the cladogram was generated using the online LefSe project2.

### Statistical analysis

Statistical analysis was carried out using STATISTICA 7.0 for Windows (StatSoft Inc., Tulsa, OK, USA). All data were presented as mean ± SD and analyzed by one-way analysis of variance (ANOVA). Duncan’s multiple range test and critical ranges was used to test differences among individual means. Difference were regarded as significant when *P* < 0.05.

## Supplementary information

**Additional file 1: Table S1.** Statistics of sequencing data of each sample after filtration. **Table S2.** Statistics of OTU species of samples on various levels. **Table S3.** Statistics of OTU clustering results of samples on various levels. **Figure S1.** Effective sequence length distribution.

## Data Availability

All data generated or analyzed during this study are included in this published article and its additional files, as well as in the NCBI Short Read Archive (SRA), under accession numbers SAMN13379773, SAMN13379774, SAMN13379775, SAMN13379776, SAMN13379777, SAMN13379778, SAMN13379779, SAMN13379780, SAMN13379781, SAMN13379782, SAMN13379783, SAMN13379784 and SAMN13379785. The datasets in this study are available from the corresponding author under on reasonable request.

## Abbreviations

DHA: Docosahexaenoic Acid
EAA: Essential amino acid
EPA: Eicosapentaenoic Acid
FM: Fishmeal
LEfSe: Linear discriminant analysis Effect Size
NMDS: Non-metric multidimensional scaling
one-way ANOVA: One-way analysis of variance
PCR: The polymerase chain reaction
RDP: The ribosomal database project classifier
SD: Standard deviation

## References

[1] Ley RE, Lozupone CA, Hamady M, Knight R, Gordon JI (2008). Worlds within worlds: evolution of the vertebrate gut microbiota. Nat Rev Microbiol.

[2] Verschuere L, Rombaut G, Sorgeloos P, Verstraete W (2000). Probiotic bacteria as biological control agents in aquaculture. Microbiol Mol Biol Rev.

[3] Nayak SK (2010). Role of gastrointestinal microbiota in fish. Aquac Res.

[4] Dhanasiri AK, Brunvold L, Brinchmann MF, Korsnes K, Bergh Ø, Kiron V (2011). Changes in the intestinal microbiota of wild Atlantic cod *Gadus morhua* L. upon captive rearing. Microb Ecol.

[5] Ringø E, Olsen RE, Mayhew TM, Myklebust R (2003). Electron microscopy of the intestinal microflora of fish. Aquaculture.

[6] Ringø E, Sperstad S, Myklebust R, Refstie S, Krogdahl A (2006). Characterisation of the microbiota associated with intestine of Atlantic cod (*Gadus morhua* L.): the effect of fish meal, standard soybean meal and a bioprocessed soybean meal. Aquaculture.

[7] Schwab C, Cristescu B, Northrup JM, Stenhouse GB, Ganzle M (2011). Diet and environment shape fecal bacterial microbiota composition and enteric pathogen load of grizzly bears. PLoS One.

[8] Uchii K, Matsui K, Yonekura R, Tani K, Kenzaka T, Nasu M, Kawabata Z (2006). Genetic and physiological characterization of the intestinal bacterial microbiota of bluegill (*Lepomis macrochirus*) with three different feeding habits. Microb Ecol.

[9] Ward NL, Steven B, Penn K, Methe BA, Deteich WH (2009). Characterization of the intestinal microbiota of two Antarctic notothenioid fish species. Extremophiles.

[10] Bemis WE, Findeis EK, Grande L (1997). An overview of Acipenseriformes. Environ Biol Fish.

[11] Ludwig A (2008). Identification of Acipenseriform species in trade. J Appl Ichthyol.

[12] Hoseinifar SH, Ringø E, Masouleh AS, Esteban MA (2016). Probiotic, prebiotic and synbiotic supplements in sturgeon aquaculture: a review. Rev Aquac.

[13] Xue M, Yun B, Wang J, Sheng H, Zheng Y, Wu X, Qin Y, Li P (2012). Performance, body compositions, input and output of nitrogen and phosphorus in Siberian sturgeon, *Acipenser baerii* Brandt, as affected by dietary animal protein blend replacing fishmeal and protein levels. Aquac Nutr.

[14] Shen L, Shi Y, Zou YC, Zhou XH, Wei QW (2014). Sturgeon aquaculture in China: status, challenge and proposals based onnation-wide surveys of 2010–2012. J Appl Ichthyol.

[15] Zhang T, Zhang P, Zhang LZ, Wang B, Gao LJ, Xia YH, Tia MP (2009). Effects of initial feeding on the growth, survival, and body biochemical composition of Siberian sturgeon (*Acipenser baerii*) larvae. Chin J Appl Ecol.

[16] Yun B, Xue M, Wang J, Sheng H, Zheng Y, Wu X, Li J (2014). Fishmeal can be totally replaced by vegetable protein blend at two protein levels in diets of juvenile Siberian sturgeon, *Acipenser baerii* Brandt. Aquac Nutr.

[17] Mohseni M, Hassani MHS, Pourali FH, Pourkazemi M, Bai SC (2011). The optimum dietary carbohydrate / lipid ratio can spare protein in growing beluga *Huso huso*. J Appl Ichthyol.

[18] Bour HAM, Esmaeili M, Kenari AA (2018). Growth performance, muscle and liver composition, blood traits, digestibility and gut bacteria of beluga (*Huso huso*) juvenile fed different levels of soybean meal and lactic acid. Aquac Nutr.

[19] Jami E, Israel A, Kotser A, Mizrahi I (2013). Exploring the bovine rumen bacterial community from birth to adulthood. ISME J.

[20] Zhang MS, Shi MH, Fan MY, Xu SH, Li YM, Zhang TX (2018). Comparative analysis of gut microbiota changes in Père David's deer populations in Beijing Milu Park and Shishou, Hubei Province in China. Front Microbiol.

[21] Sekirov I, Russell SL, Antunes LCM, Finlay BB (2010). Gut microbiota in health and disease. Physiol Rev.

[22] Xu GL, Xing W, Li TL, Ma ZH, Liu CX, Jiang N, Luo L (2018). Effects of dietary raffinose on growth, non-specific immunity, intestinal morphology and microbiome of juvenile hybrid sturgeon (*Acipenser baeri* Brandt ♀ × *A*. *schrenckii* Brandt ♂). Fish Shellfish Immunol.

[23] Lv SJ, Zhao W, Shi ZG, Wang S, Wei J (2018). Comparative study of the intestinal microbial community of wild and cultured Kaluga sturgeon, *Huso dauricus*. Aquac Res.

[24] Geraylou Z, Souffreau C, Rurangwa E, De Meester L, Courtin CM, Delcour JA, Buyse J, Ollevier F (2013). Effects of dietary arabinoxylan-oligosaccharides (AXOS) and endogenous probiotics on the growth performance, non-specific immunity and gut microbiota of juvenile Siberian sturgeon (*Acipenser baerii*). Fish Shellfish Immunol.

[25] Navarrete P, Magne F, Araneda C, Fuentes P, Barros L, Opazo R (2012). PCR-TTGE analysis of 16S rRNA from rainbow trout (*Oncorhynchus mykiss*) gut microbiota reveals host-specific communities of active bacteria. PLoS One.

[26] Zhao LL, Wang G, Siegel P, He C, Wang HZ, Zhao WJ (2013). Quantitative genetic background of the host influences gut microbiomes in chickens. Sci Rep.

[27] Li TT, Long M, Gatesoupe FJ, Zhang QQ, Li AH, Gong XN (2015). Comparative analysis of the intestinal bacterial communitiesin different species of carp by pyrosequencing. Microb Ecol.

[28] Hsu HY, Chang FC, Wang YB, Chen SH, Lin YP, Lin CY, Han YS (2018). Revealing the compositions of the intestinal microbiota of three Anguillid eel species using 16S rDNA sequencing. Aquac Res.

[29] Li J, Ni J, Li J, Wang C, Li X, Wu S, Zhang T, Yu Y, Yan Q (2014). Comparative study on gastrointestinal microbiota of eight fish species with different feeding habits. J Appl Microbiol.

[30] Catalán N, Villasante A, Wacyk J, Ramírez C, Romero J (2018). Fermented soybean meal increases lactic acid bacteria in gut microbiota of Atlantic Salmon (*Salmo salar*). Probiotics Antimicrob Proteins.

[31] Llewellyn MS, Boutin S, Hoseinifar SH, Derome N (2014). Teleost microbiomes: the state of the art in their characterization, manipulation and importance in aquaculture and fisheries. Front Microbiol.

[32] Wen J, Sun XF (2009). Research progress on intestinal microecological regulation of aquatic animals. Feed Res.

[33] Wang WJ, Pan BH, Sun DY, Sun XF, Li C (2012). The formation and physiological function of intestinal flora of aquatic animals. Feed Res.

[34] Miao SY, Zhao CZ, Zhu JY, Hu JT, Dong XJ, Sun LS (2018). Dietary soybean meal affects intestinal homoeostasis by altering the microbiota, morphology and infammatory cytokine gene expression in northern snakehead. Sci Rep.

[35] Bruce TJ, Neiger RD, Brown ML (2018). Gut histology, immunology and the intestinal microbiota of rainbow trout, *Oncorhynchus mykiss* (Walbaum), fed process variants of soybean meal. Aquac Res.

[36] Torrecillas S, Mompel D, Caballero MJ, Montero D, Merrifield D, Rodiles A, Robaina L, Zamorano MJ, Karalazos V, Kaushik S, Izquierdo M (2017). Effect of fishmeal and fish oil replacement by vegetable meals and oils on gut health of European sea bass (*Dicentrarchus labrax*). Aquaculture.

[37] Klase G, Lee S, Liang S, Kim J, Zo YG, Lee J (2019). The microbiome and antibiotic resistance in integrated fishfarm water: implications of environmental public health. Sci Total Environ.

[38] Zhang J, Yang Y, Zhao L, Li Y, Xie S, Liu Y (2015). Distribution of sediment bacterial and archaeal communities in plateau freshwater lakes. Appl Microbiol Biotechnol.

[39] Li XH, Yu YH, Li C, Yan QY (2018). Comparative study on the gut microbiotas of four economically important Asian carp species. Sci China Life Sci.

[40] Xu J, Bjursell MK, Himrod J, Deng S, Carmichael LK, Chiang HC (2003). A genomic view of the human-*bacteroides thetaiotaomicron* symbiosis. Science.

[41] Bäckhed F, Ley RE, Sonnenburg JL, Peterson DA, Gordon JI (2005). Host-bacterial mutualism in the human intestine. Science.

[42] Xu Q, Yuan XY, Gu TT, Li Y, Dai WC, Shen XK (2017). Comparative characterization of bacterial communities in geese fed all-grass or high-grain diets. PLoS One.

[43] Matsui H, Kato Y, Chikaraishi T, Moritani M, Ban-Tokuda T, Wakita M (2009). Microbial diversity in ostrich cecaas revealed by 16S ribosomal RNA gene clone library and detection of novel *Fibrobacter* species. Anaerobe.

[44] Pope PB, Denman SE, Jones M, Tringe SG, Barry K, Malfatti SA, McHardy AC, Cheng JF, Hugenholtz P, McSweeney CS, Morrison M (2010). Adaptation to herbivory by the Tammar wallaby includes bacterial and glycoside hydrolase profiles different from other herbivores. Proc Natl Acad Sci U S A.

[45] Bäckhed F, Ding H, Wang T, Hooper LV, Koh GY, Nagy A, Semenkovich CF, Gordon JI (2004). The gut microbiota as an environmental factor that regulates fat storage. Proc Natl Acad Sci U S A.

[46] Bäckhed F, Manchester JK, Semenkovich CF, Gordon JI (2007). Mechanisms underlying the resistance to diet-induced obesity in germ-free mice. Proc Natl Acad Sci U S A.

[47] Ley RE, Bäckhed F, Turnbaugh P, Lozupone CA, Knight RD, Gordon JI (2005). Obesity alters gut microbial ecology. Proc Natl Acad Sci U S A.

[48] Turnbaugh PJ, Ley RE, Mahowald MA, Magrini V, Mardis ER, Gordon JI (2006). An obesity-associatedgut microbiome with increased capacity for energy harvest. Nature.

[49] Ye L, Amberg J, Chapman D, Gaikowski M, Liu WT (2014). Fish gut microbiota analysis diferentiates physiology and behavior of invasive Asian carp and indigenous American fish. ISME J.

[50] Saha S, Roy RN, Sen SK, Ray AK (2006). Characterization of cellulose-producing bacteria from the digestive tract of tilapia, *Oreochromis mossambica* (Peters) and grass carp, *Ctenopharyngodon idella* (Valenciennes). Aquac Res.

[51] Hu X, Yu J, Wang C, Chen H (2014). Cellulolytic bacteria associated with the gut of *Dendroctonus armandi* larvae (Coleoptera: Curculionidae: Scolytinae). Forests.

[52] Liu H, Guo XW, Gooneratne R, Lai RF, Zeng C, Zhan FB (2016). The gut microbiome and degradation enzyme activity of wild freshwater fishes infuenced by their trophic levels. Sci Rep.

[53] Wu S, Wang G, Angert ER, Wang W, Li W, Zou H (2012). Composition, diversity, and origin of the bacterial community in grass carp intestine. PLoS One.

[54] Liu CH, Chiu CH, Wang SW, Cheng W (2012). Dietary administration of the probiotic, Bacillus subtilis E20, enhances the growth,innate immune responses, and disease resistance of the grouper, *Epinephelus coioides*. Fish Shellfish Immunol.

[55] Hao K, Wu ZQ, Li DL, Yu XB, Wang GX, Ling F (2017). Effects of dietary administration of *Shewanella xiamenensis* A-1, *Aeromonas veronii* A-7, and *Bacillus subtilis*, single or combined, on the grass carp (*Ctenopharyngodon idella*) intestinal microbiota. Probiotics Antimicrob Proteins.

[56] González-Félixa ML, Gatlin DM, Urquidez-Bejarano P, Reé-Rodríguez C, Duarte-Rodríguez L, Sánchez F, Casas-Reyes A, Yamamoto FY, Ochoa-Leyvac A, Perez-Velazquez M (2018). Effects of commercial dietary prebiotic and probiotic supplements on growth, innate immune responses, and intestinal microbiota and histology of *Totoaba macdonaldi*. Aquaculture.

[57] Wu SG, Gao TH, Zheng YZ, Wang WW, Cheng YY, Wang GT (2010). Microbial diversity of intestinal contents and mucus in yellow catfish (*Pelteobagrus fulvidraco*). Aquaculture.

[58] Standen B, Rodiles A, Peggs D, Davies S, Santos G, Merrifield D (2015). Modulation of the intestinal microbiota and morphology of tilapia, *Oreochromis niloticus*, following the application of a multi-species probiotic. Appl Microbiol Biotechnol.

[59] Caporaso JG, Kuczynski J, Tombaugh J, Bittinger K, Bushman FD, Costello EK, Fierer N, Pena AG, Goodrich JK, Gordon JI (2010). QIIME allows analysis of high-throughput community sequencing data. Nat Methods.

[60] Edgar RC (2010). Search and clustering orders of magnitude faster than BLAST. Bioinformatics.

[61] Caporaso JG, Bittinger K, Bushman FD, Desantis TZ, Andersen GL, Knight R (2010). PyNAST: a flexible tool for aligning sequences to a template alignment. Bioinformatics.

[62] Desantis TZ, Hugenholtz P, Larsen N, Rojas M, Brodie EL, Keller K, Huber T, Dalevi D, Hu P, Andersen GL (2006). Greengenes, a chimera-checked 16S rRNA gene database and workbench compatible with ARB. Appl Environ Microbiol.

[63] Wang Q, Garrity GM, Tiedje JM, Cole JR (2007). Naive Bayesian classifier for rapid assignment of rRNA sequences into the new bacterial taxonomy. Appl Environ Microbiol.

[64] Schloss PD, Westcott SL, Ryabin T, Hall JR, Hartmann M, Hollister EB, Lesniewski RA, Oakley BB, Parks DH, Robinson CJ, Sahl JW, Stres B, Thallinger GG, Van Horn DJ, Weber CF (2009). Introducing Mothur: open-source, platform-independent, community-supported software for describing and comparing microbial communities. Appl Environ Microbiol.

