# Supplementary material for: Comparative study on the effects of different feeding habits and diets on intestinal microbiota in Acipenser baeri Brandt and Huso huso
Source: BMC Microbiol. 2019 Dec 16;19:297. doi: 10.1186/s12866-019-1673-6 (PMC6915974; doi:10.1186/s12866-019-1673-6)
Supplement: Supplementary file 1 — Additional file 1: Table S1. Statistics of sequencing data of each sample after filtration. Table S2. Statistics of OTU species of samples on various levels. Table S3. Statistics of OTU clustering results of samples on various levels. Figure S1. Effective sequence length distribution. [file 12866_2019_1673_MOESM1_ESM.docx]

**Supplementary Data**

**Table S1** Statistics of sequencing data of each sample after ﬁltration

| Sample | Raw_Tags | Clean_Tags | Final_tags | Effective (%) |
| --- | --- | --- | --- | --- |
| BL1 | 23046 | 18670 | 14350 | 62.27 |
| BL2 | 31153 | 25582 | 19327 | 62.04 |
| BL3 | 24178 | 21635 | 15716 | 65.00 |
| BL4 | 21189 | 19401 | 14491 | 68.39 |
| BL5 | 39809 | 27683 | 21736 | 54.60 |
| BH1 | 25182 | 24048 | 19253 | 76.45 |
| BH2 | 23034 | 21840 | 17417 | 75.61 |
| BH3 | 34341 | 28083 | 21665 | 63.09 |
| BH4 | 39753 | 35536 | 27231 | 58.50 |
| BH5 | 23929 | 22038 | 17568 | 73.42 |
| SH1 | 34464 | 32848 | 28143 | 81.66 |
| SH2 | 19168 | 18217 | 13740 | 71.68 |
| SH3 | 24495 | 21769 | 16063 | 65.58 |

Sample, name of sequencing sample; Raw_Tags, number of original sequences obtained by splicing paired-endedreads; Clean_Tags, number of optimized sequences after ﬁltering the original sequence; Final_tags, number of tag corresponding to each sample in the final OTU table; Effective (%), proportion of effective tags among Raw_Tags =100 × (Final_tags / Raw_Tags).

**Table S2** Statistics of OTU species of samples on various levels

| Sample | Kimdom | Phylum | Class | Order | Family | Genus | Species |
| --- | --- | --- | --- | --- | --- | --- | --- |
| BL1 | 1 | 13 | 20 | 38 | 66 | 101 | 28 |
| BL2 | 1 | 13 | 23 | 46 | 85 | 135 | 35 |
| BL3 | 1 | 10 | 17 | 36 | 74 | 118 | 32 |
| BL4 | 1 | 13 | 22 | 41 | 78 | 128 | 34 |
| BL5 | 1 | 12 | 20 | 39 | 72 | 114 | 33 |
| BH1 | 1 | 13 | 20 | 42 | 76 | 124 | 32 |
| BH2 | 1 | 14 | 21 | 41 | 78 | 121 | 33 |
| BH3 | 1 | 15 | 21 | 43 | 80 | 111 | 27 |
| BH4 | 1 | 14 | 25 | 51 | 94 | 150 | 41 |
| BH5 | 1 | 12 | 22 | 46 | 87 | 132 | 41 |
| SH1 | 1 | 9 | 17 | 36 | 70 | 103 | 28 |
| SH2 | 1 | 13 | 21 | 40 | 73 | 102 | 28 |
| SH3 | 1 | 12 | 22 | 40 | 70 | 118 | 31 |

**Table S3** Statistics of OTU clustering results of samples on various levels

| Sample | Kimdom | Phylum | Class | Order | Family | Genus | Species |
| --- | --- | --- | --- | --- | --- | --- | --- |
| BL1 | 14306 | 9174 | 9024 | 8993 | 8983 | 6366 | 2967 |
| BL2 | 19322 | 18543 | 18463 | 17722 | 17657 | 13773 | 7519 |
| BL3 | 15714 | 15050 | 15009 | 14561 | 14503 | 10537 | 5021 |
| BL4 | 14490 | 14064 | 14023 | 13602 | 13284 | 10734 | 5094 |
| BL5 | 21710 | 9308 | 7928 | 7707 | 7677 | 5357 | 2339 |
| BH1 | 19253 | 19219 | 19143 | 19038 | 18960 | 13519 | 8416 |
| BH2 | 17412 | 17228 | 17153 | 17015 | 16851 | 13336 | 9684 |
| BH3 | 21616 | 10528 | 10289 | 10198 | 10160 | 7790 | 3163 |
| BH4 | 27231 | 27198 | 27124 | 27039 | 26671 | 22985 | 7373 |
| BH5 | 17568 | 17549 | 17465 | 17435 | 17273 | 14729 | 11258 |
| SH1 | 28134 | 27988 | 27948 | 27653 | 27423 | 21329 | 12465 |
| SH2 | 13740 | 13734 | 13541 | 13399 | 13364 | 10453 | 4544 |
| SH3 | 16063 | 16056 | 16037 | 16029 | 15686 | 13077 | 6035 |


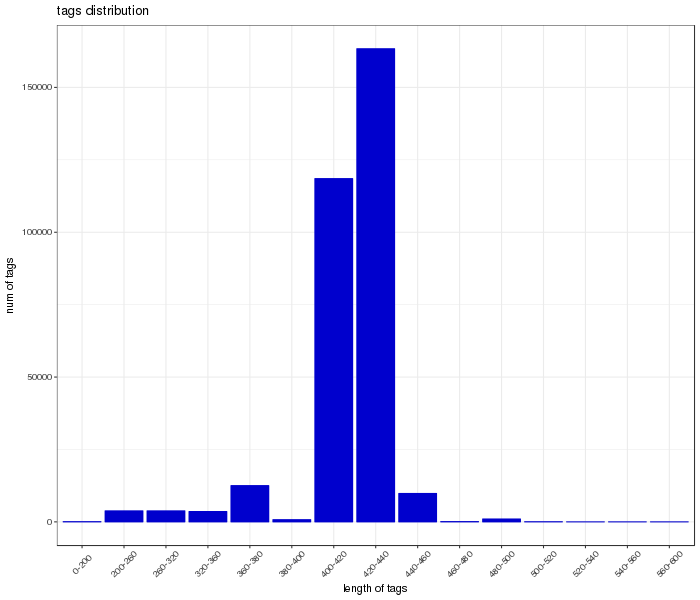


**Fig. S1** Effective sequence length distribution. The x-coordinate is the sequence length range; the y-coordinate is the number of tags
